# Phylogenetic insights into the diversity of *Chryseobacterium* species

**DOI:** 10.1099/acmi.0.000019

**Published:** 2019-05-03

**Authors:** Shivakumara Siddaramappa, Anushree Narjala, Vandana Viswanathan, Chaitra Maliye, Raghavendran Lakshminarayanan

**Affiliations:** 1 Institute of Bioinformatics and Applied Biotechnology, Biotech Park, Electronic City, Bengaluru 560100, Karnataka, India

**Keywords:** *Bacteroidetes*, *Flavobacteriaceae*, *Chryseobacterium*, B-14859, WG4, Flexirubin

## Abstract

The genus *Chryseobacterium* was formally established in 1994 and contains 112 species with validly published names. Most of these species are yellow or orange coloured, and contain a flexirubin-type pigment. The genomes of 83 of these 112 species have been sequenced in view of their importance in clinical microbiology and potential applications in biotechnology. The National Center for Biotechnology Information taxonomy browser lists 1415 strains as members of the genus *Chryseobacterium *, of which the genomes of 94 strains have been sequenced. In this study, by comparing the 16S rDNA and the deduced proteome sequences, at least 20 of these strains have been proposed to represent novel species of the genus *Chryseobacterium *. Furthermore, a yellow-coloured bacterium isolated from dry soil in the USA (and identified as *Flavobacterium* sp. strain B-14859) has also been reconciled as a novel member of the genus *Chryseobacterium* based on the analysis of 16S rDNA sequences and the presence of flexirubin. Yet another bacterium (isolated from a water sample collected in the Western Ghats of India and identified as *Chryseobacterium* sp. strain WG4) was also found to represent a novel species. These proposals need to be validated using polyphasic taxonomic approaches.

## Introduction

*Chryseobacterium* was circumscribed as a novel genus of the family *Flavobacteriaceae* by Vandamme *et al*. [1] to provide a separate taxonomic status for six members of the genus *Flavobacterium* that appeared to be distantly related to the type species *Flavobacterium aquatile *. This characterization was based on DNA:rRNA hybridization and chemotaxonomic studies [1]. The name of the genus (*chryseos*=golden) was due to the fact that the bacteria produced yellow to orange-coloured colonies on solid media [1], and it was reported that the pigment was flexirubin [1, 2]. [* Flavobacterium *] *gleum*, which was isolated by Holmes *et al*. [3] from human clinical specimens, was designated as the type species of *Chryseobacterium* [1]. With *Chryseobacterium meningosepticum* being distinct from other bacteria within the group [1], it appears that *Chryseobacterium* was destined to be heterogeneous since the time of its inception. The genus *Elizabethkingia* was later carved out of *Chryseobacterium* to accommodate *C. meningosepticum* [4]. Although there were a mere 18 species of *Chryseobacterium* in 2006 [5], that number had risen to 58 by 2014 [6]. At the time of writing (January 2019), the list of prokaryotic names with standing in nomenclature (www.bacterio.net) contained 112 *Chryseobacterium* spp. with validly published names, representing every letter of the English alphabet except Q. Many of these species were reported to be multidrug resistant [5]. In view of their importance in clinical microbiology and potential applications in biotechnology, the genomes of 83 *Chryseobacterium* spp. have been sequenced and are available in National Center for Biotechnology Information (NCBI) (www.ncbi.nlm.nih.gov/genome/?term=Chryseobacterium). Despite this wealth of data, only a few systematic attempts have been made to compare these genomes [7–10]. Notable among these attempts is the quest to characterise antibiotic resistance and identify the genetic basis for the same [7, 8, 10]. Furthermore, using genome-based taxonomic analysis, it has been proposed that the genus *Chryseobacterium* be emended to include members of the closely related genus *Epilithonimonas* [11]. At the time of writing (January 2019), the NCBI taxonomy browser (www.ncbi.nlm.nih.gov/Taxonomy/Browser/wwwtax.cgi?id=59732) had listed 1415 strains as members of the genus *Chryseobacterium *. The genomes of 94 of these strains have also been sequenced and are available in NCBI (www.ncbi.nlm.nih.gov/genome/genomes/13849?). However, very little is known about the sources or characteristics of these strains, and it is likely that many of them belong to one of the 112 species already described. The objectives of this study were to extend the current knowledge about the diversity of *Chryseobacterium* spp., and provide insights into the taxonomic status of strains that are not yet assigned to a species within the genus.

## Methods

### Phylogenetic analysis using CVTree3

Phylogenetic analysis using the web server CVTree3, which is an alignment- and parameter-free method that relies on the oligopeptide content (K-tuple length) of conserved proteins to deduce evolutionary relatedness [12], was performed as described previously [13].

Briefly, the deduced proteome sequences (excluding plasmid-encoded proteins) of *Chryseobacterium* spp. were downloaded from UniProt (www.uniprot.org/proteomes/). The protein sequences were saved as multifasta files with the extension .faa. The multifasta files for each strain were uploaded on to the CVTree3 web server (http://tlife.fudan.edu.cn/cvtree/cvtree/) and analysed by selecting all available K-tuple length options (from 3 to 9). Since the best *K*-values for bacteria were shown to be 5–6 [12], the proteome tree was visualised at *K*=6. The output from CVTree3 was saved as a Newick file, and the tree was rendered using the Interactive Tree Of Life (iTOL) web server version 4 (https://itol.embl.de/).

### Phylogenetic analysis using mega 7.0

Pairwise alignments of DNA sequences were performed using ClustalW with default parameters. The pairwise distance matrix derived from these alignments was used to construct a guide tree by the neighbour-joining method. Subsequent progressive alignment was based on the guide tree. Phylogeny was reconstructed using the maximum likelihood method (with 1000 bootstrap replicates) and the Tamura–Nei substitution model in mega 7.0. The output from mega was saved as a Newick file, and the tree was rendered using iTOL.

### PCR, cloning and sequencing

Bacterial genomic DNA was isolated using the snap-chill method. Briefly, a loopful of fresh bacterial culture was resuspended in 100 µl sterile ddH_2_O in a 1.5 ml microcentrifuge tube. The cell suspension was boiled in a water bath for 10 min. The boiled cell suspension was incubated at −80 °C for 10 min. The frozen suspension was thawed and centifuged (~18 600 ***g*** for 10 min at 4 °C) using a Hettich MIKRO 220 R centrifuge. The supernatant was transferred to a sterile 0.5 ml microcentrifuge tube and used in PCR after serial dilution. Amplification of the 16S rDNA was performed using the 27F (5′-AGAGTTTGATCMTGGCTCAG-3′) and 1492R (5′-TACGGYTACCTTGTTACGACTT-3′) primers. PCR products (~1.5 kb) were gel purified using the GeneJET Gel Extraction Kit (Thermo Scientific) and ligated into the pTZ57R/T vector (InsTAclone PCR Cloning Kit, Thermo Scientific). Competent cells of *Escherichia coli* DH5α were prepared using the CaCl_2_ method and transformed with the ligated products. Transformants were selected on Luria–Bertani (LB) agar plates containing ampicillin (100 µg ml^−1^) and recombinants were selected by blue-white screening. Recombinants were confirmed by plasmid purification (GeneJET Plasmid Miniprep Kit, Thermo Scientific) and restriction digestion. DNA inserts within pTZ57R/T were sequenced using the Sanger sequencing method.

### Pigment extraction and analysis

Strain B-14859 was procured from the Agricultural Research Service Culture Collection (Peoria, Illinois, USA), which is the only known official source of the bacterium. This bacterium was cultured using LB medium at 30 °C with aeration (shaking at 200 r.p.m.). Solid LB medium was prepared using 2 % agar for culturing bacteria from glycerol stocks or plating broth cultures to test purity. Biochemical tests were performed based on the descriptions in the VetBact online resource (www.vetbact.org/) of the Faculty of Veterinary Medicine and Animal Science of the Swedish University of Agricultural Sciences. Liquid bacterial cultures were pelletted in sterile Oak Ridge tubes using a fixed angle rotor (JA20, ~12000 ***g*** for 10 min at 4 °C) in an Avanti J-25 centrifuge (Beckman Coulter, USA). Wet biomass (80 or 160 mg) was obtained from the pellets and resuspended in 1 ml acetone by gentle vortexing. The suspension was lysed using a VCX 750 Vibra-Cell sonicator (Sonics and Materials, USA) for 10 min (30 % amplitude with 5 s on/off pulse). The yellow-coloured supernatant was collected by centrifugation and scanned using a Cary 100 UV-Vis spectrophotometer (Agilent Technologies). Spectra in the 200–800 nm wavelength range were recorded.

## Results and discussion

### Identification of novel species using 16S rDNA sequences

A total of 33 strains that were not yet assigned to a species within the genus *Chryseobacterium* were chosen for further analysis based on the availability of their genome sequences in the public databases. For 29 of these strains, 16S rDNA sequences were obtained from GenBank (Table 1). The closest homologues of these sequences were searched within the ‘16S ribosomal RNA sequences (Bacteria and Archaea)’ database of NCBI using blastn. The output was optimised by selecting the ‘Highly similar sequences (megablast)’ option. From this search, the top hits (those with the highest blastn score, having a query coverage of >90 %) for each sequence were recorded (Table 1). To characterise each strain further, the criteria proposed by Chun *et al*. [14] were used. If the identity of the top hit was ≥98.7 %, then the strain was inferred not to represent a novel species. For four strains (AG844, CBo1, ERMR1 : 04 and YR203), the top hits had 99–100 % identity with blast scores >2600 (Table 1). It is very likely these strains belong to *C. cucumeris *, *C. formosense *, *C. polytrichastri* and *C. vrystaatense *, respectively.

**Table 1.** Annotation of strains not yet assigned to a species within the genus *Chryseobacterium
*

Strain name	GenBank accession no. of 16S rDNA sequence	Top hit obtained from blastn of 16S rDNA sequence (strain no. provided in parenthesis)	GenBank accession no. of 16S rDNA sequence of the top hit	blast score	Query coverage (%)	Identity (%)	Inference based on blastn analysis
P1-3	MK095748	*Chryseobacterium gallinarum* (100)	NR_133726	2529	92	99	Closely related to *C. gallinarum*
52	CLU96_0011	*Chryseobacterium luteum* (P 456/04)	NR_042596	2551	96	98	New species
AG844	MK095749	*Chryseobacterium cucumeris* (GSE06)	NR_156145	2601	93	99	Closely related to *C. cucumeris*
BLS98	ACM40_01015	*Chryseobacterium oranimense* (H8)	NR_044168	2543	95	99	Closely related to *C. oranimense*
CBo1	MK095750	*Chryseobacterium formosense* (CC-H3-2)	NR_036872	2678	97	100	Closely related to *C. formosense*
CF314	MH818596	*Chryseobacterium polytrichastri* (YG4-6)	NR_134710	2459	97	97	New species
ERMR1 : 04	KT766029	*Chryseobacterium polytrichastri* (YG4-6)	NR_134710	2636	98	99	Closely related to *C. polytrichastri*
FH1	JX293123	*Chryseobacterium halperniae* (H1)	NR_115989	2534	100	98	New species
FH2	KU736794	*Chryseobacterium scophthalmum* (LMG 13028)	NR_025386	2499	97	98	New species
FP211-J200	MK095752	*Chryseobacterium halperniae* (H1)	NR_115989	2577	97	98	New species
HMWF028	MK095753	*Chryseobacterium culicis* (R4-1A)	NR_117008	2152	97	99	Closely related to *C. culicis*
HMWF035	MK095754	*Chryseobacterium gambrini* (5-1St1a)	NR_042505	2586	92	99	Closely related to *C. gambrini*
Hurlbut01	None	None	None	None	None	None	None
IHB B 17019	KP208630	*Chryseobacterium taihuense* (THMBM1)	NR_109542	2486	99	97	New species
ISE14	EU034659	*Chryseobacterium lactis* (KC1864)	NR_126256	2505	98	99	Closely related to *C. lactis*
JAH	KU855373	*Chryseobacterium piscium* (LMG 23089)	NR_042410	2484	98	97	New species
JM1	JX293122	*Chryseobacterium oleae* (CT348)	NR_134002	2591	99	99	Closely related to *C. oleae*
Leaf180	MK095755	*Chryseobacterium gregarium* (P 461/12)	NR_042647	2444	97	97	New species
Leaf201	MK095756	*Chryseobacterium gregarium* (P 461/12)	NR_042647	2575	97	98	New species
Leaf394	None	None	None	None	None	None	None
Leaf404	MK095757	*Chryseobacterium echinoideorum* (CC-CZW010)	NR_145657	2471	99	97	New species
Leaf405	MK095758	*Chryseobacterium piscium* (LMG 23089)	NR_042410	2562	98	98	New species
MOF25P	MK095759	*Chryseobacterium balustinum* (NBRC 15053)	NR_113721	2165	96	99	Closely related to *C. balustinum*
MYb7	KU902431	*Chryseobacterium lactis* (KC1864)	NR_126256	2580	99	99	Closely related to *C. lactis*
OV279	MK095760	*Chryseobacterium oleae* (CT348)	NR_134002	2584	100	99	Closely related to *C. oleae*
PMSZPI	JF768716	*Chryseobacterium culicis* (R4-1A)	NR_117008	2534	100	99	Closely related to *C. culicis*
RU33C	MK095761	*Chryseobacterium culicis* (R4-1A)	NR_117008	2558	97	98	New species
RU37D	MK095762	*Chryseobacterium daeguense* (K105)	NR_044069	2573	98	98	New species
SCN 40–13	None	None	None	None	None	None	None
StRB126	AB581546	*Chryseobacterium jejuense* (JS17-8)	NR_044300	2290	100	99	Closely related to *C. jejuense*
T16E-39	CEY12_12625	*Chryseobacterium xinjiangense* (TSBY-67)	NR_131771	2553	100	98	New species
YR203	MK095763	*Chryseobacterium vrystaatense* (R-23566)	NR_042370	2708	96	99	Closely related to *C. vrystaatense*
YR221	None	None	None	None	None	None	None

A strain can be predicted to represent a novel species if the identity of the top hit is <98.7 % [14]. For five strains, the identities of the top hits were 97 % (with blast scores >2444). For nine strains, the identities of the top hits were 98 % (with blast scores >2499). Pending further confirmation, these 14 strains were deemed to represent novel *Chryseobacterium* spp. (Table 1). For four other strains (Hurlbut01, Leaf394, SCN 40–13 and YR221), 16S rDNA sequences were not available in GenBank (Table 1). It appears that the 16S rDNA genes of these strains were not covered during genome sequencing because even annotation using RAST (http://rast.nmpdr.org/) did not reveal them. However, the unavailability of 16S rDNA sequences was not a major handicap since these strains could be characterised using their genomes or other phylogenetic markers.

### Identification of novel species using deduced proteome sequences

**Fig. 1.**
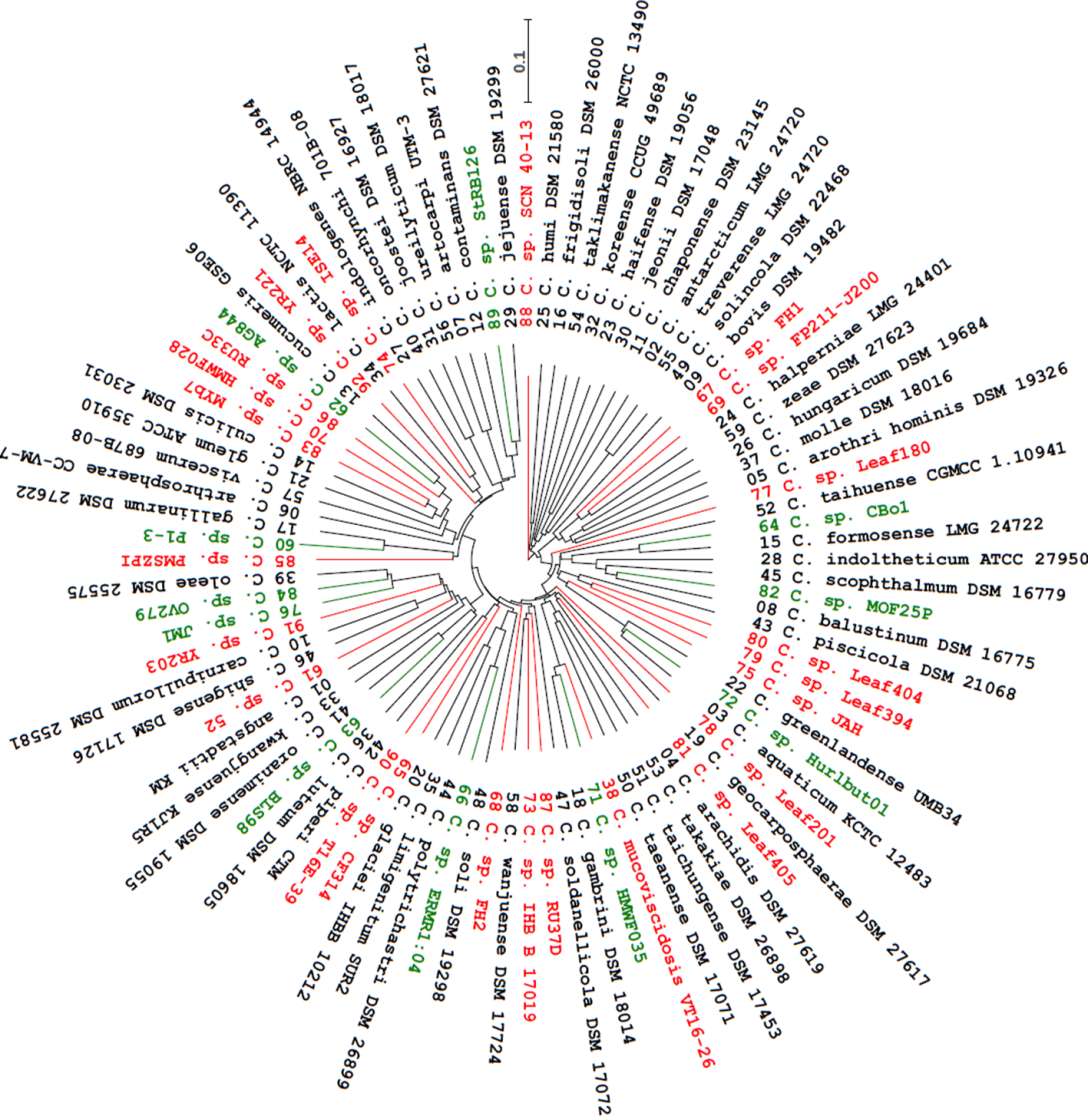
Phylogenetic tree based on proteome sequences. The tree was constructed using the neighbour-joining method by the web server CVTree3, and visualised at *K*=6. Numbers at the end of each branch refer to the serial numbers in Table 2. Black lines and text indicate *Chryseobacterium* spp. with validly published names (*n*=58). Green line and text indicate strains that were predicted not to represent novel species (*n*=11). Red lines and text indicate strains that were predicted to represent novel species (*n*=23). The proteome of *Flavobacterium columnare* ATCC 49512 (UniProt Proteome ID: UP000005638) was used as the outgroup, which does not appear in the figure. The tree was scaled based on branch length values. The bar allows the estimation of branch lengths (e.g. strain SCN 40–13 has a branch length of 0.2235, which is approximately 2.235 times the length of the bar).

Previously, Chun *et al*. [14] had shown that analysis of 16S rDNA sequences could be combined with whole genome comparisons to correctly identify and recognise novel species. On the same principles, proteome sequence-based analyses were performed using CVTree3 to check the relationships among 92 strains (including the 33 listed in Table 1) of *Chryseobacterium* spp. (Table 2). The phylogenetic tree (Fig. 1) derived from this analysis indicated that the genus *Chryseobacterium* is diverse and polyphyletic. In total, 16 of the 21 species previously analysed using whole genome sequences by Hahnke *et al*. [11] were present in this tree (Table 2 and Fig. 1). Although the methods of analyses are different, the branching patterns of these 16 species were similar in Fig. 1 and in the tree reported by Hahnke *et al*. [11]. For example, *C. antarcticum *, *C. jeonii *, *C. koreense* and *C. solincola* were located on a major branch in both trees. *Chryseobacterium bovis *, which was shown to cluster with *Epilithonimonas tenax* by Hahnke *et al*. [11], was found on a separate branch containing five other *Chryseobacterium* spp. (Fig. 1). Furthermore, *C. angstadtii *, *C. kwangjuense* and *C. luteum* occurred on yet another major branch, as did *C. gallinarum *, *C. gleum* and *C. indologenes* (Fig. 1). Hahnke *et al*. [11] showed that *C. aquaticum* and *C. greenlandense* were closely related to each other and co-locate with *C. formosense *. A similar outcome was conspicuous in the proteome sequence-based tree (Fig. 1).

**Table 2.** Characteristics of 59 *Chryseobacterium* spp. and 33 strains not yet assigned to species within the genus

Serial no.	Species	Strain	Taxonomy ID	Genome accession	Proteome ID	Predicted proteins	Colour*	Pigment*	References
1	* C. angstadtii *†	KM‡	558151	LFND01000000	UP000036261	4285	Yellow–orange	Flexirubin	[15]
2	* C. antarcticum *†	LMG 24720‡	266748	JPEP01000000	UP000028349§	2660	Yellow	Not reported	[16]
3	* C. aquaticum *†	KCTC 12483‡	452084	LLYZ01000000	UP000051682	3362	Yellow	Flexirubin	[17]
4	*C. arachidis*	DSM 27619‡	1416778	FQUT01000000	UP000184518§	4490	Yellow	Flexirubin	[18]
5	*C. arothri*	DSM 19326‡	420404	FNWX01000000	UP000198555§	2619	Yellow	Not reported	[19]
6	*C. arthrosphaerae*	CC-VM-7‡	651561	MAYG01000000	UP000093432	4443	Yellow	Not reported	[20]
7	*C. artocarpi*	UTM-3‡	1414727	MAYH01000000	UP000092651§	4306	Yellow	Flexirubin	[21]
8	*C. balustinum*	DSM 16775‡	246	FUZE01000000	UP000190669	4472	Yellow	Flexirubin	[1]
9	* C. bovis *†	DSM 19482‡	1121284	FTPU01000000	UP000187261§	3210	Yellow	Flexirubin and carotenoid	[22]
10	*C. carnipullorum*	DSM 25581‡	1124835	FRCD01000000	UP000184354	4673	Yellow	Flexirubin	[23]
11	*C. chaponense*	DSM 23145‡	713588	FTOI01000000	UP000185839§	2771	Yellow	Not reported	[24]
12	*C. contaminans*	DSM 27621‡	1423959	FRBM01000000	UP000184069	4213	Yellow	Flexirubin	[25]
13	*C. cucumeris*	GSE06‡	1813611	LUVZ01000000	UP000075550§	4621	Yellow	Not reported	[26]
14	*C. culicis*	DSM 23031‡	680127	FNWQ01000000	UP000198561	4402	Yellow	Flexirubin	[27]
15	* C. formosense *†	LMG 24722‡	236814	JPRP01000000	UP000028713§	3694	Yellow	Flexirubin	[28]
16	*C. frigidisoli*	DSM 26000‡	1125876	FOQT01000000	UP000198931§	2760	Yellow	Not reported	[29]
17	* C. gallinarum *†	DSM 27622‡	1324352	CP009928	UP000035213§	3813	Yellow	Flexirubin	[25]
18	*C. gambrini*	DSM 18014‡	373672	FTOV01000000	UP000185781§	4438	Yellow	Flexirubin	[30]
19	*C. geocarposphaerae*	DSM 27617‡	1416776	PGFD01000000	UP000228740	3685	Yellow	Flexirubin	[18]
20	*C. glaciei*	IHBB 10212‡	1685010	CP015199	UP000077824§	4228	Yellow to orange	Flexirubin	[31, 32]
21	* C. gleum *†	ATCC 35910‡	525257	ACKQ02000000	UP000002969	5279	Yellow	Flexirubin	[3]
22	* C. greenlandense *†	UMB34‡	345663	LMAI01000000	UP000054388	3588	Orange	Flexirubin	[33]
23	*C. haifense*	DSM 19056‡	1450526	JASZ02000000	UP000197587§	2680	Yellow	Flexirubin and carotenoid	[34]
24	*C. halperniae*	LMG 24401‡	421072	JPLY01000000	UP000028623	3493	Orange	Flexirubin	[11, 35]
25	*C. humi*	DSM 21580‡	493375	FNUS01000000	UP000236738§	2872	Yellow	Not reported	[36]
26	*C. hungaricum*	DSM 19684‡	454006	FNBH01000000	UP000199203§	3591	Yellow	Flexirubin	[37]
27	* C. indologenes *†	NBRC 14944‡	1218103	BAVL01000000	UP000019060§	4192	Yellow	Flexirubin	[38]
28	*C. indoltheticum*	ATCC 27950‡	254	FTMF01000000	UP000185725	3885	Yellow	Flexirubin	[1]
29	*C. jejuense*	DSM 19299‡	445960	FNEG01000000	UP000199426	4577	Yellow	Flexirubin	[39]
30	* C. jeonii *†	DSM 17048‡	266749	JSYL01000000	UP000031473	2813	Yellow	Not reported	[16]
31	*C. joostei*	DSM 16927‡	112234	FTNZ01000000	UP000186106	4491	Yellow	Flexirubin	[40]
32	* C. koreense *†	CCUG 49689‡	1304281	LFNG01000000	UP000035900	2784	Yellow	Not reported	[41, 42]
33	* C. kwangjuense *†	KJ1R5‡	267125	LPUR01000000	UP000070513	4445	Yellow	Not reported	[43]
34	*C. lactis*	NCTC 11390‡	1241981	PPEH01000000	UP000236262	4897	Yellow	Not reported	[44]
35	*C. limigenitum*	SUR2‡	1612149	FPKW01000000	UP000182034§	4308	Yellow	Flexirubin	[45]
36	* C. luteum *†	DSM 18605‡	421531	JPRO01000000	UP000028703§	4032	Orange	Flexirubin	[46]
37	*C. molle*	DSM 18016‡	216903	FRAM01000000	UP000184498§	3334	Pale-cream	Not reported	[30]
38	*C. mucoviscidosis*	VT16-26‡	1945581	MVAG01000000	UP000196355	4041	Not reported	Not reported	[47]
39	*C. oleae*	DSM 25575‡	491207	FOVD01000000	UP000198769	4753	Yellow to orange	Flexirubin	[48]
40	*C. oncorhynchi*	701B-08‡	741074	PPEI02000000	UP000236182§	4323	Pale orange	Flexirubin	[49]
41	*C. oranimense*	DSM 19055‡	421058	FQWT01000000	UP000184047	4261	Orange	Flexirubin	[50]
42	* C. piperi *†	CTM‡	558152	JPRJ01000000	UP000028709§	3502	Yellow–orange	Flexirubin	[51]
43	*C. piscicola*	DSM 21068‡	551459	FTOJ01000000	UP000186246§	3092	Yellow	Flexirubin	[52]
44	*C. polytrichastri*	DSM 26899‡	1302687	FRAV01000000	UP000184364	4630	Yellow	Flexirubin	[53]
45	*C. scophthalmum*	DSM 16779‡	59733	FSRQ01000000	UP000184782	4013	Orange	Flexirubin	[54]
46	*C. shigense*	DSM 17126‡	297244	FTNY01000000	UP000186373	4421	Yellow	Flexirubin	[55]
47	*C. soldanellicola*	DSM 17072‡	311333	FNKL01000000	UP000199627§	3680	Yellow	Not reported	[56]
48	* C. soli *†	DSM 19298‡	445961	JPRH01000000	UP000028705§	4064	Yellow	Flexirubin	[39]
49	* C. solincola *†	DSM 22468‡	510955	JSYK01000000	UP000031275§	2087	Yellow	Not reported	[57]
50	*C. taeanense*	DSM 17071‡	311334	FNDW01000000	UP000198869§	3464	Yellow	Not reported	[56]
51	*C. taichungense*	DSM 17453‡	295069	FOBV01000000	UP000199450§	4057	Yellow	Flexirubin	[58]
52	*C. taihuense*	CGMCC 1.10941‡	1141221	FNHD01000000	UP000199242§	3186	Orange-yellow	Flexirubin	[59]
53	*C. takakiae*	DSM 26898‡	130268	FQVO01000000	UP000184236§	3767	Yellow	Flexirubin	[60]
54	*C. taklimakanense*	NCTC 13490‡	536441	LT906465	UP000215196§	2590	Pale yellow	Not reported	[44, 61]
55	*C. treverense*	DSM 22251‡	631455	FORQ01000000	UP000242560	2170	Yellow	Flexirubin	[62]
56	*C. ureilyticum*	DSM 18017‡	373668	FTOL01000000	UP000186744	4747	Yellow	Flexirubin	[30]
57	*C. viscerum*	687B-08‡	1037377	PPEG02000000	UP000236413	5025	Pale orange	Flexirubin	[63]
58	*C. wanjuense*	DSM 17724‡	356305	FOIU01000000	UP000199469§	4159	Yellow	Flexirubin	[64]
59	*C. zeae*	DSM 27623‡	1416779	FSRK01000000	UP000185207§	3663	Yellow	Flexirubin	[18]
60	* Chryseobacterium* sp.	P1-3	1517683	JPEQ01000000	UP000028723	3118	Not reported	Not reported	[65]
61	* Chryseobacterium* sp.	52	2035213	PEEX01000000	UP000231109§	4668	Unknown	Unknown	None
62	* Chryseobacterium* sp.	AG844	2183998	QGTV01000000	UP000246445	4406	Unknown	Unknown	None
63	* Chryseobacterium* sp.	BLS98	885586	LFNF01000000	UP000036030§	3836	Unknown	Unknown	None
64	* Chryseobacterium* sp.	CBo1	1869230	MAUH01000000	UP000093224	4001	Unknown	Unknown	None
65	* Chryseobacterium* sp.	CF314||	1144316	AKJY01000000	UP000007509§	4113	Yellow	Flexirubin	[66]
66	* Chryseobacterium* sp.	ERMR1 : 04	1705393	LIRF01000000	UP000037945	4510	Yellow	Not reported	[67]
67	* Chryseobacterium* sp.	FH1	1233951	JPLZ01000000	UP000028641§	3367	Unknown	Unknown	None
68	* Chryseobacterium* sp.	FH2	1674291	LFNE01000000	UP000036315§	3519	Unknown	Unknown	None
69	* Chryseobacterium* sp.	FP211-J200	1792309	LSHB01000000	UP000077158	3747	Yellow	Not reported	[68]
70	* Chryseobacterium* sp.	HMWF028	2056862	QAJA01000000	UP000244693	4131	Unknown	Unknown	None
71	* Chryseobacterium* sp.	HMWF035	2056868	QEHP01000000	UP000245732	4497	Unknown	Unknown	None
72	* Chryseobacterium* sp.	Hurlbut01	1681828	LGIP01000000	UP000036769§	3267	Not reported	Not reported	[69]
73	* Chryseobacterium* sp.	IHB B 17019	1721091	CP013293	UP000058667§	3633	Unknown	Unknown	None
74	* Chryseobacterium* sp.	ISE14||	475075	PPED02000000	UP000236594§	4626	Yellow	Not reported	[70–72]
75	* Chryseobacterium* sp.	JAH	1742858	LMAH01000000	UP000054026	3479	Unknown	Unknown	None
76	* Chryseobacterium* sp.	JM1	1233950	JPRN01000000	UP000028731	4364	Unknown	Unknown	None
77	* Chryseobacterium* sp.	Leaf180	1736289	LMPJ01000000	UP000051405§	3021	Unknown	Unknown	None
78	* Chryseobacterium* sp.	Leaf201	1735672	LMKA01000000	UP000051097§	3820	Unknown	Unknown	None
79	* Chryseobacterium* sp.	Leaf394	1736361	LMQH01000000	UP000051822§	3828	Unknown	Unknown	None
80	* Chryseobacterium* sp.	Leaf404	1736366	LMQM01000000	UP000051183	3502	Unknown	Unknown	None
81	* Chryseobacterium* sp.	Leaf405	1736367	LMQN01000000	UP000051197§	3994	Unknown	Unknown	None
82	* Chryseobacterium* sp.	MOF25P	1664318	LFEG01000000	UP000092708§	4239	Unknown	Unknown	None
83	* Chryseobacterium* sp.	MYb7	1827290	PCOU01000000	UP000238576	4535	Unknown	Unknown	None
84	* Chryseobacterium* sp.	OV279	1500285	FQUD01000000	UP000184187	4748	Unknown	Unknown	None
85	* Chryseobacterium* sp.	PMSZPI	1033900	PIZV01000000	UP000233651§	3251	Unknown	Unknown	None
86	* Chryseobacterium* sp.	RU33C	1907398	FTMT01000000	UP000186612	4161	Unknown	Unknown	None
87	* Chryseobacterium* sp.	RU37D	1907397	FTMM01000000	UP000185978	2783	Unknown	Unknown	None
88	* Chryseobacterium* sp.	SCN 40–13	1660093	MEDR01000000	UP000094247§†	4288	Unknown	Unknown	None
89	* Chryseobacterium* sp.	StRB126	878220	AP014624	UP000031650	4811	Not reported	Not reported	[73]
90	* Chryseobacterium* sp.	T16E-39	2015076	CP022282	UP000198250§	4288	Not reported	Not reported	[74]
91	* Chryseobacterium* sp.	YR203	1500291	FQVE01000000	UP000184108	4963	Not reported	Not reported	[75]
92	* Chryseobacterium* sp.	YR221	1500293	FWXM01000000	UP000192341	4386	Not reported	Not reported	[75]

*Colour and pigment as reported in the references listed in the last column.

†These species were previously analysed using whole genome sequences by Hahnke *et al*. [11].

‡Type strain.

§Reference proteomes (UniProt defines these as ‘well-studied model organisms and other organisms of interest for biomedical research and phylogeny’).

||Strains CF314 and ISE14 were proposed to represent novel species after the analysis reported here were completed.

Strains AG844, CBo1 and ERMR1 : 04 were predicted not to represent novel species based on 16S rDNA sequence comparisons (Table 1). These strains had shorter branches and clustered with *C. cucumeris *, *C. formosense* and *C. polytrichastri *, respectively, in the proteome sequence-based tree (Fig. 1). Strain YR203 had a longer and distinct branch in the tree because the top hit for this strain (* C. vrystaatense *, Table 1) was not included in the analysis. Among other strains predicted not to represent novel species (Table 1), P1-3, BLS98, HMWF035, JM1, MOF25P and StRB126 also had relatively short branches and clustered with *C. gallinarum *, *C. oranimense *, *C. gambrini *, *C. olae*, *C. balustinum* and *C. jejuense *, respectively (Fig. 1). However, although strain ISE14 was predicted to be closely related to *C. lactis* (Table 1), it actually had a longer branch and clustered with *C. indologenes* (Fig. 1). Similarly, although strain MYb7 was predicted to be closely related to *C. lactis* (Table 1), it occurred in a cluster with strain HMWF028 and *C. culicis* (Fig. 1). Surprisingly, strain PMSZPI clustered with *C. gallinarum* (Fig. 1), although it was predicted to be closely related to *C. culicis* (Table 1). These discrepancies could be due to misidentified strains and/or their sequences. Further analysis are required to establish the phylogenetic status and novelty of strains HMWF028, ISE14, MYb7 and PMSZPI.

All five strains (CF314, IHB B 17019, JAH, Leaf180 and Leaf404) that were deemed to represent new species based on the comparison of their 16S rDNA sequences (top hits having an identity of 97 %, blast scores >2444, Table 1) had relatively longer branches (Fig. 1). More importantly, all nine strains (52, FH1, FH2, FP211-J200, Leaf201, Leaf405, RU33C, RU37D and T16E-39) whose top hits had an identity of 98 % (blast scores >2499, Table 1) also had longer branches (Fig. 1). Among these 14 strains, Leaf405 shared a branch with *C. arachidis *, T16E-39 shared a branch with *C. piperi* and was located close to CF314, IHB B 17019 shared a branch with *C. wanjuense* and was located close to RU37D, FP211-J200 shared a branch with *C. halperniae* and was located close to FH1 (Fig. 1). Among the four strains lacking 16S rDNA sequences (Table 1), SCN 40–13 and YR221 appear to represent novel species based on the length and distinctness of the branches on which they are located in the tree (Fig. 1). Leaf394 shared a branch with Leaf404 and may also represent novel species. However, Hurlbut01 may not represent a novel species and is closely related to *C. aquaticum* (Fig. 1). Using whole genome comparisons, Tetz and Tetz [47] had proposed *C. mucoviscidosis* to be a novel species. The deduced proteome sequence-based tree provides further credence to this proposal, and shows that *C. mucoviscidosis* is related to *C. gambrini* (Fig. 1).

### Characterization of strain NRRL B-14859

It has been more than two decades since Hou [76] identified and described a Gram-negative, non-motile, rod-shaped bacterium that produced yellowish-brown colonies. This bacterium (referred to as strain NRRL B-14859, also known as strain DS5) was identified as a member of the genus *Flavobacterium* [76]. This strain was shown to produce oxygenated fatty acids such as 10-ketostearic acid and 10-hydroxystearic acid using oleic acid [76] and vegetable oils [77] as substrates. It was also shown to convert linoleic acid to 10-hydroxy-12(Z)-octadecenoic acid [78]. The bioconversion of oleic acid by this strain was reported to be more efficient than the bioconversion of linoleic acid. More importantly, the oleate hydratase of strain B-14859 was predicted to be a C-10 positional-specific enzyme with a preference for 18-carbon mono-unsaturated fatty acid [79]. This strain was further characterised in the context of results reported in the previous two sections.

**Fig. 2.**
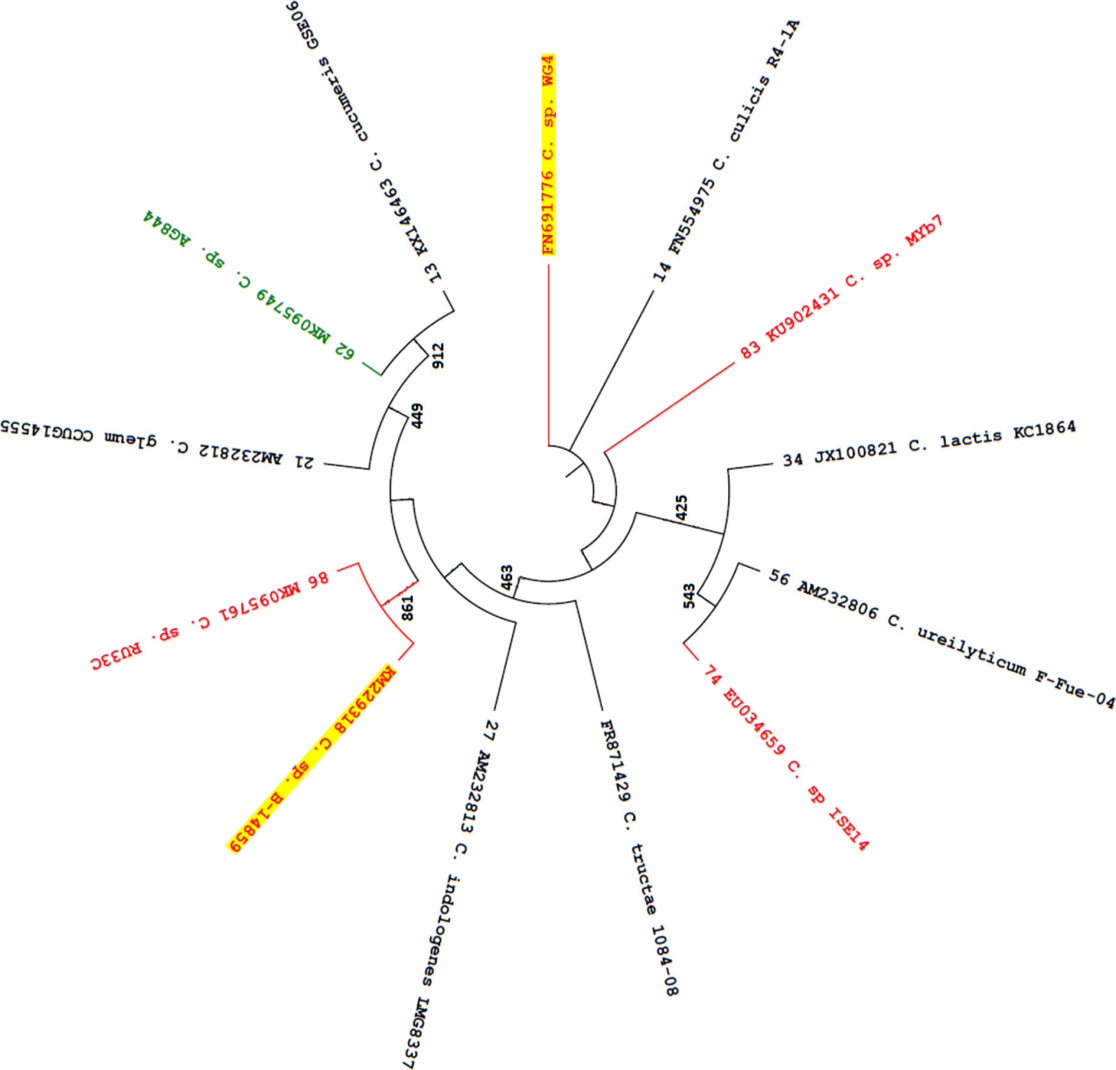
Phylogenetic tree based on 16S rDNA sequences. The analysis involved 13 sequences and the tree was constructed using the maximum likelihood method in mega 7.0. Bootstrap values of 1000 replicates are indicated as numbers at the nodes (only values >400 are shown). The tree was rooted and rendered without using branch lengths. Numbers at the end of each branch, when present, refer to the strains as in Table 2. Black lines and text indicate *Chryseobacterium* spp. with validly published names (*n*=7). Green line and text indicate strain AG844 that was predicted to be very closely related to *C. cucumeris* GSE06 in Fig. 1. Red lines and text indicate five strains that were predicted to represent novel species (only three of these are shown in Fig. 1); strains B-14859 and WG4 lacked genome sequences and are shaded.

Golden-yellow-coloured colonies of strain B-14859 were seen on LB agar plates after overnight incubation at 30 °C (the strain could also grow at 20 or 42 °C). Strain B-14859 was resistant to ampicillin (100 µg ml^−1^), kanamycin (50 µg ml^−1^), tetracycline (30 µg ml^−1^) and spermidine (15 µg ml^−1^), but lacked plasmids. It was catalase, urease and gelatinase positive, but was oxidase and indole negative. The 16S rDNA gene of strain B-14859 was cloned and sequenced. The closest homologues of this sequence were retrieved as described in the first section. The top five hits (from *C. ureilyticum *, *C. indologenes *, *C. gleum *, *Chryseobacterium bernardetii* and *C. vrystaatense *) had 97–98 % identity (with a blast score of 2471–2508, query coverage of 96–99 %). Therefore, based on the inferences drawn in Table 1, it appeared that strain B-14859 belongs to the genus *Chryseobacterium *, and represents a novel species. To further characterise the taxonomic position of this strain, phylogenetic analysis was performed using 16S rDNA sequences (961 bp). In the phylogenetic tree, *C. cucumeris* and *C. gleum* clustered on a main branch with strains AG844 and RU33C (Fig. 2). A similar clustering was also observed in the proteome sequence-based tree (Fig. 1). Since strains B-14859 and RU33C were located on a sub-branch within this main branch (Fig. 2), it is likely that they are closely related. Furthermore, *Chryseobacterium* sp. strain WG4, which was isolated from a water sample collected in the Western Ghats of India [80], was located on a separate branch (Fig. 2) and may also represent a novel species. Notably, *C. lactis* clustered on a main branch with *C. ureilyticum* (and strain ISE14) in the 16S rDNA sequence-based tree (Fig. 2), but with *C. indologenes* (and strain ISE14) in the proteome sequence-based tree (Fig. 1). Interestingly, strain MYb7, which was predicted to be closely related to *C. lactis* (Table 1), but clustered with *C. culicis* in the proteome sequence-based tree (Fig. 1), was located on a separate branch in Fig. 2. As indicated previously, further analyses are required to resolve the taxonomic position of strains ISE14 and MYb7.

**Fig. 3.**
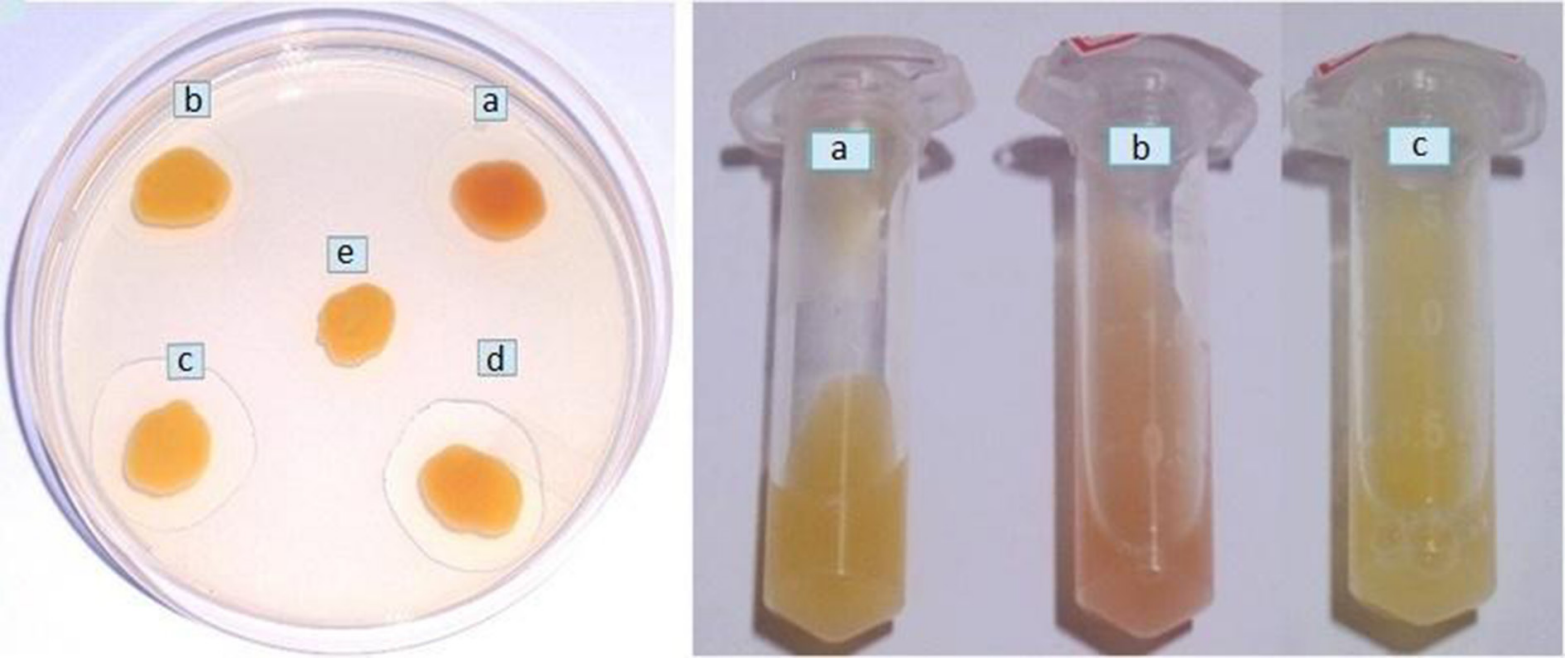
Reversible colour change of pigment of strain B-14859. Left: colour changes to red when 3 % KOH added to culture spot (a); no change in colour when 1.5 N HCl is added (b); no change in colour when 1.5 N HCl is added first, followed by 3 % KOH (c); colour changes from red to yellow when 3 % KOH is added first, followed by 1.5 N HCl (d); control culture spot (e). Right: control culture (a); colour changes to red when 3 % KOH is added to the broth culture (b); colour changes from red to yellow when 3 % KOH is added first, followed by 1.5 N HCl (c).

**Fig. 4.**
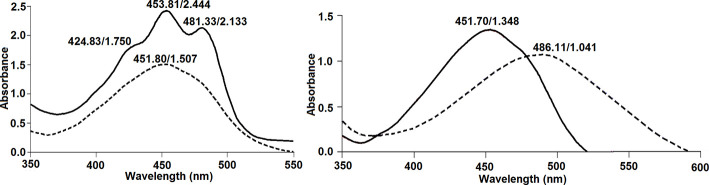
Absorption spectra of pigments. Left: the absorption spectrum of pigments extracted using acetone from strain B-14859 shows a single peak (broken line), whereas that of *S. paucimobilis* strain NRRL B-54 shows three characteristic peaks (solid line). Right: the ~451 nm peak (solid line) of pigments extracted using acetone from strain B-14859 showed a bathochromic shift (broken line) after the addition of 20 % KOH. In all spectra, the numbers indicate wavelength/peak absorbance.

Overnight cultures of strain B-14859 on solid medium or in broth had a distinct fruity odour. Interestingly, fruity odour was also reported in *C. indologenes* [38], but not in *C. gleum* [3]. Based on the characterization of strain WG4 [80], it is likely that the fruity aroma of *Chryseobacterium* sp. is due to ethyl-2-methylbutyrate and ethyl-3-methylbutyrate. Yabuuchi *et al*. [38] reported that the flexirubin-type pigment of *C. indologenes* turned deep red after one drop of 3 % potassium hydroxide (KOH) solution was added, and that the colour change was reversed when one drop of 1.5 N hydrochloric acid (HCl) was added. A similar result was obtained with the pigment of strain B-14859 (Fig. 3). Yabuuchi *et al*. [38] also reported that the absorption spectra of pigments extracted using acetone from three strains of *C. indologenes* had a single peak at ~451 nm. The pigment of strain B-14859 extracted using acetone showed a similar peak (Fig. 4). In contrast, the UV-Vis absorption spectrum of acetone-extracted pigments of *Sphingomonas paucimobilis* strain B-54, which produces C_40_ carotenoids, showed three peaks (Fig. 4). Furthermore, the ~451 nm peak of strain B-14859 shifted to a higher wavelength after the addition of 20 % KOH (Fig. 4), as reported previously for *C. indologenes* strains [38]. Therefore, it is likely that the pigment of strain B-14859 is of the flexirubin type. Flexirubins, first identified in *Flexibacter elegans* [81], are polyene compounds that are insoluble in many organic solvents or water [82]. The biological functions of these pigments, which appear to be pervasive in *Chryseobacterium* spp. (Table 2), are yet to be characterised.
